# Supplementary material for: Screening for α-Glucosidase-Inhibiting Saponins from Pressurized Hot Water Extracts of Quinoa Husks
Source: Foods. 2022 Sep 29;11(19):3026. doi: 10.3390/foods11193026 (PMC9563573; doi:10.3390/foods11193026)
Supplement: Supplementary file 1 [file foods-11-03026-s001.zip › foods-1904440-supplementary.pdf]

Table S1. The response surface analysis of variance (ANOVA).

Source	Sum of Source	df	Mean Source	F-value	P-value
Model	62.93	9	6.99	52.48	0.0001
A-Flow Rate	1.89	1	1.89	14.17	0.0070
B-Temperature	10.72	1	10.72	80.45	0.0001
C-Time	0.032	1	0.032	0.24	0.6390
AB	4.09	1	4.09	30.70	0.0009
AC	2.12	1	2.12	15.88	0.0053
BC	2.70	1	2.70	20.30	0.0028
A^2	0.34	1	0.34	2.57	0.1531
B^2	29.78	1	29.78	223.53	0.0001
C^2	13.09	1	13.09	98.23	0.0001
Residual	0.93	7	0.13		
Lack of Fit	0.40	3	0.13	1.00	0.4792
Error	0.53	4	0.13		
Cor Total	63.87	16			

Table S2. Affinity values of compounds 1-23 and acarbose with α-glucosidase.

Compounds number	Affinity (kcal/mol)	Compounds number	Affinity (kcal/mol)
1	−8.6	13	−8.3
2	−8.1	14	-12.6
3	−9.7	15	−7.2
4	-9.4	16	-12.2
5	-7.1	17	-8.7
6	-9.3	18	-8.6
7	-9.4	19	-12.7
8	-8.9	20	-8.7
9	-11.6	21	-12.6
10	−8.3	22	-9.2
11	-9.4	23	-9.4
12	−7.7	acarbose	-8.5
